# Apis Mellifica: Its Poison

**Published:** 1882-04

**Authors:** James Heddon

**Affiliations:** Dowagiac, Mich.


### APIS MILLIFICA: ITS POISON.

JAMES HEDDON, DOWAGIAC, MICH.

At the request of Dr. Ballard, of Chicago, I herewith give a brief account of my peculiar experience with the poison from the honey bee.

I have been a specialist in apiculture, for the past fourteen years, and have learned something considerable about the business; have consequently neglected learning other things, among which anatomy and materia medica are conspicuous. You will, consequently, please accept my plain statements given in the parlance of an unprofessional.

I am 37 years years old, nervous, sanguine temperament (in the extreme), weigh 135 lbs., and have good health, but excessive general nervous irritation, mostly in the form of mild chronic neuralgia. My voice has *always* indicated rather weak bronchial organs. Never had a cough, however, before I experienced bee-poisoning. I began bee-keeping on a somewhat extensive scale, in the year 1878. As stated before, it has been my sole occupation ever since. I have had as many as 550 colonies in three apiaries at one time.

Seven years ago, I began to notice an itching sensation in the ears. This would come on at times, and after about two years, it extended to the glands inside the mouth, and near the root of the tongue. After about one more year, the sensation began to be very severe in the roof of the mouth, just around and in front of the palate. It was at this time that I first discovered that the affection had a connection with the bees. To sweep the floor of one of my rooms, where bees had fallen and been trodden upon, was sure to bring on this sensation *at once.* Next, I found, that to open a hive and breath the odor of the bees (especially if not thoroughly subdued) would also cause the trouble. But, business must be attended to, and I persisted in working among the bees and bee-hives, till the itching and tingling sensation crept down the bronchial tubes all around about the lungs. One night after a day's work among the bees, I woke up about midnight with the asthma.

A celebrated travelling doctor, examined me "free," and gave me some medicine for $10.00, and told me I had a case of "bronchial" asthma, that looked wicked. He looked at my throat (shortly after a bee had) and "must have something done for it at once." I was not sure *then* that bee-poison was the cause.

Finally, I began making tests; leaving the whole business for two weeks, I was almost entirely clear of all, except the first symptoms in the ears, which only troubled me occasionally.

The first breath of bee-poison I inhaled, on my return, was followed by all the former symptoms, seemingly in an increased degree, and in ten minutes my throat turned red, and clearly showed severe irritation. I resolved to hire more help, add to the business of honey production that of manufacturing and selling bee-keepers' supplies, and in that way absent myself from contact with the virus to a greater extent, and yet keep busy.

I have done so, and am in consequence quite free from the trouble most of the time. But if I at any time come in contact with the poison, my symptoms seem to be as radical as ever, yes, even *more* so. I will cite one instance. All apiarists know that often when a maddened threatening bee flits around one's head she generally discharges into the air her poison. It is recognized by the nasel organs only. Now, I have found that this occurs when none of the five senses of the healthy bee-master recognizes it.

One day last autumn, after I had kept from all contact with the poison for some weeks, and had no troublesome symptoms, I stepped into my yard, when an ugly bee passed within about eight inches of my face, discharging poison as he passed. About one half-hour after I was seized with perhaps the most severe paroxysm of my experience. First, symptoms were an almost unbearable itching, tingling sensation of the roof of the mouth, and so on down the breathing tubes as far as they extend; then an asthmatic filling-up sensation.

For more than eight hours I could not speak aloud. For two or three days I could not raise my voice above common conversation. All passed off, leaving me as well as ever, by keeping away from the poison.

In correspondence with one Italian and one German, of large apicultural experience in the old countries, I learn that such cases are known there.

When we bear in mind the fact that the older system of honey production, as practiced in the old world, and in this country till recently, did not bring the operator into any such near or constant contact with the bees, and that cases where individuals in this country, working upon the improved system, for any such length of time as fourteen years as a specialty, are very rare, we have reason to look for the development of many more such cases as my own.

Dr. Ballard expresses the desire to doctor my case upon the homœopathic system; to which I assent, knowing, as I think I do, that the homœopathic treatment possesses that splendid feature over all other schools, that if it *don't* cure it don't *kill*. I will here say publicly, what I said by letter to him, that "faith" will play no part in any possible cure of my case.

I know that the laws of hygiene point strongly to the claimed base principle of Homœopathy, but I have *always* failed to get *any* effect from homœopathic remedies, given by professed thoroughbreds. I can at most consider the science as one only vaguely understood at *best*. Still its successful operations force all the other schools to step down on a level with it, to say the least, in all observing and thinking minds.

Any questions bearing upon the case, will be answered promptly and with pleasure, for I think I may safely say in the name of our fraternity, that a specific remedy for these symptoms, coming from any school, will put us under many obligations to that school.

